# Cathodic Polarization Coats Titanium Based Implant Materials with Enamel Matrix Derivate (EMD)

**DOI:** 10.3390/ma7032210

**Published:** 2014-03-14

**Authors:** Matthias J. Frank, Martin S. Walter, Marina Rubert, Bernd Thiede, Marta Monjo, Janne E. Reseland, Håvard J. Haugen, Ståle Petter Lyngstadaas

**Affiliations:** 1 Department of Biomaterials, Institute for Clinical Dentistry, University of Oslo, P.O. Box 1109 Blindern, Oslo NO-0317, Norway; E-Mails: m.j.frank@odont.uio.no (M.J.F.); m.s.walter@odont.uio.no (M.S.W.); marta.monjo@uib.es (M.M.); j.e.reseland@odont.uio.no (J.E.R.); s.p.lyngstadaas@odont.uio.no (S.P.L.); 2 Institute of Medical and Polymer Engineering, Technische Universität München, Boltzmannstrasse 15, Garching 85748, Germany; 3 Department of Fundamental Biology and Health Sciences, Research Institute on Health Sciences (IUNICS), University of Balearic Islands, Palma de Mallorca ES-07122, Spain; E-Mail: marinarubert@gmail.com; 4 The Biotechnology Centre of Oslo, University of Oslo, P.O. Box 1125 Blindern, Oslo NO-0317, Norway; E-Mail: bernd.thiede@biotek.uio.no

**Keywords:** bioactive coating, coating technique, enamel matrix derivate, EMD, titanium, titanium-zirconium, surface modification

## Abstract

The idea of a bioactive surface coating that enhances bone healing and bone growth is a strong focus of on-going research for bone implant materials. Enamel matrix derivate (EMD) is well documented to support bone regeneration and activates growth of mesenchymal tissues. Thus, it is a prime candidate for coating of existing implant surfaces. The aim of this study was to show that cathodic polarization can be used for coating commercially available implant surfaces with an immobilized but functional and bio-available surface layer of EMD. After coating, XPS revealed EMD-related bindings on the surface while SIMS showed incorporation of EMD into the surface. The hydride layer of the original surface could be activated for coating in an integrated one-step process that did not require any pre-treatment of the surface. SEM images showed nano-spheres and nano-rods on coated surfaces that were EMD-related. Moreover, the surface roughness remained unchanged after coating, as it was shown by optical profilometry. The mass peaks observed in the matrix-assisted laser desorption/ionization time-of-flight mass spectroscopy (MALDI-TOF MS) analysis confirmed the integrity of EMD after coating. Assessment of the bioavailability suggested that the modified surfaces were active for osteoblast like MC3M3-E1 cells in showing enhanced Coll-1 gene expression and ALP activity.

## 1. Introduction

Titanium based endosseous dental implants have shown improved clinical performance over recent years [1]. Although the response of bone tissue to an endosseous dental implant is governed by several factors, the implant’s surface morphology and surface chemistry largely influence the biological response to an implant [2,3]. Modifications of the surface roughness and chemistry to improve bone healing by machining, sand-blasting, acid-etching, or their combination, are common approaches for modifying the surface chemistry and for altering the surface topography and morphology on a micro- and nano-scale [4–9]. Blasting and etching have been shown to be successful in optimizing surface roughness and enhancing surface reactivity by increasing surface hydride levels for improved clinical performance [10–19]. Reducing environmental carbon contamination has been shown to further enhance the surface energy and wettability of sand-blasted and acid-etched (SBAE) surfaces. Such a reactive surface can be maintained by handling under protective cover gas and storage in saline solution [20,21]. A different approach to improve the surface by etching in hydrofluoric acid has shown elevated hydrogen levels and traces of fluoride on the surface [22,23]. Dental implants with fluoride-doped titanium dioxide have demonstrated enhanced osteoblast differentiation and bone growth [24–26].

Another, but less explored, approach in improving implant surfaces is the biochemical modification of the surface. A commonly used approach is the biomimetic deposition of hydroxyapatite (HA) on metallic surfaces [27]. Surfaces coated with HA have shown improved *in vivo* performance compared to untreated surfaces [28]. Moreover it has been shown that a HA layer may be used for incorporating biomolecules like proteins or antibiotics [29–32]. A variety of other successful biochemical surface modifications with peptides and extracellular matrix proteins and have shown that biochemically modified surfaces can improve bone healing compared to unmodified titanium surfaces [33,34]. As there is a variety of biomolecules available that promote bone healing, a surface modification by chemically attaching such a biomolecule to the surface of an implant directly offers a potential for enhancing implant performance in bone whilst maintaining the characteristics of the original implant. However, the challenges of making a bioactive coating bioavailable and to maintain its function remain [34].

Lyngstadaas and Ellingsen suggested using a polarization process to attach charged biomolecules to the surface in order to stimulate bone healing [35]. Cathodic polarization in acidic solution creates a hydride layer on titanium or titanium alloys that can be used as an activated surface for attaching charged biomolecules [35]. A previous study, which used such a process on smooth, commercially pure titanium showed that cathodic reduction of titanium in acidic solutions was successfully used in order to create thick hydride layers on the surface [36]. Our previous study showed that cathodic polarization on a SBAE surface exhibited a non-linear but cyclic development of the hydride layer [37]. Such a cyclic hydrogen development offers the potential for attaching charged biomolecules to the surface even faster than on commercially pure titanium like we have shown for strontium and doxycycline in our previous studies [38,39].

In search for a candidate biomolecule which could be attached to the surface of an implant by the proposed polarization process, enamel matrix derivate (EMD) appeared to be a promising candidate as it mainly contains amelogenins, which are the major component of the enamel extracellular matrix [40]. Lyngstadaas *et al.* [41] showed the potential of EMD for use in bone regeneration and implantology. Promising results of EMD supporting periodontal bone regeneration and the angiogenic effect of EMD have been shown in various studies [42–44]. Moreover, major components in EMD have been reported to have bipolar properties, which are a requirement for being used in an electro-coating process [45–47]. Hence, this study chose to use EMD for exploring the feasibility of a bioactive surface coating by means of the aforementioned polarization process.

The aim of this study was to show that a cathodic polarization process can be used for coating EMD onto commercially available dental implant surfaces. The secondary aim of this study was to show that EMD was bio-available and maintained its function after coating.

## 2. Results

### 2.1. Surface Chemistry

**Figure 1.**
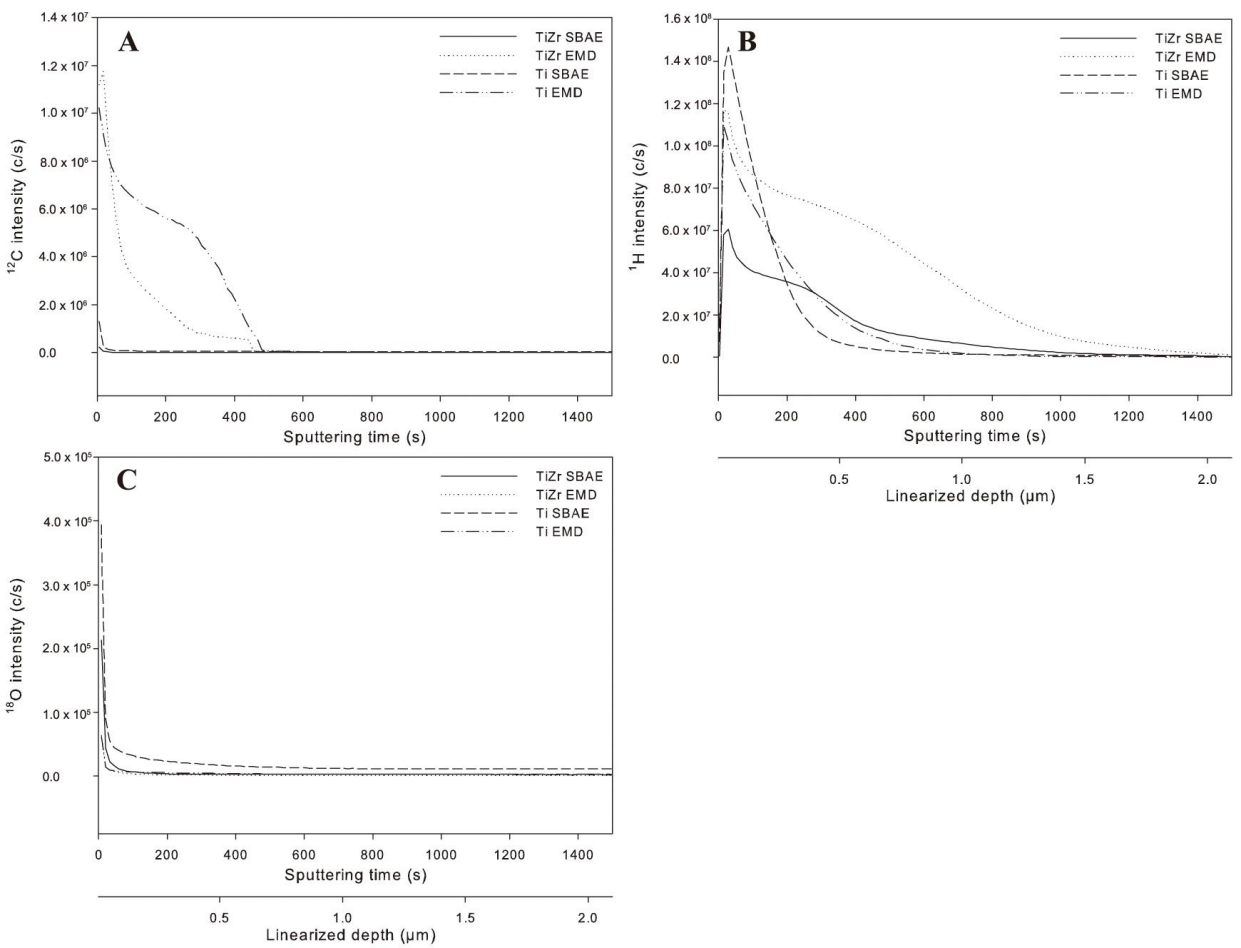
SIMS depth profiles of the ^12^C (**A**); ^1^H (**B**) and ^18^O (**C**) isotopes.

Depth profiles acquired by SIMS (Figure 1, Table 1) revealed increased carbon layer thickness, total carbon and total hydrogen content for TiZr EMD and Ti EMD compared to the respective SBAE surfaces. In detail, total carbon was increased 7.6-fold and total hydrogen was increased 2.1-fold for TiZr EMD compared to TiZr SBAE, while Ti EMD showed a 22-fold increase in total carbon and a 3.9-fold increase in hydrogen levels when compared to Ti SBAE. A similar trend was observed for the maximum intensity of hydrogen and carbon.

XPS analysis revealed that pure EMD consisted mainly of carbon (C 1s) at 60.91% (Table 2), whereas 52.72% of this carbon was present as a carbon single bond (C–C), 20.81% as a double bond (C=C), and 26.47% as a single carbon-oxygen bond (C–O) (Table 3). Oxygen (O 1s) was the second largest component of pure EMD at 24.69%, whereas 80.62% of this oxygen was bound as organic oxygen and 19.38% as a carbon-oxygen single bond (not shown in the table). In addition to carbon and oxygen, pure EMD also showed 13.9% of nitrogen (N 1s) and traces of silica (Si 2p) and sulfur (S 2p).

EMD-coated surfaces of both materials showed a shift in the binding state of the carbon on the surface towards a distribution of C–C, C=C, and C–O/C–N bonds that was comparable to the distribution observed for pure EMD. TiZr EMD and Ti EMD showed an increase in C=C bonds of *circa* 10 pp compared to the SBAE surface (Table 3). Moreover, the specific bonds of oxygen showed over 70 pp more organically bond oxygen for EMD-coated samples than for SBAE samples. While TiZr EMD and Ti EMD also showed Si and N, only TiZr EMD had S on the surface as had been observed for pure EMD.

### 2.2. Surface Morphology

**Figure 2.**
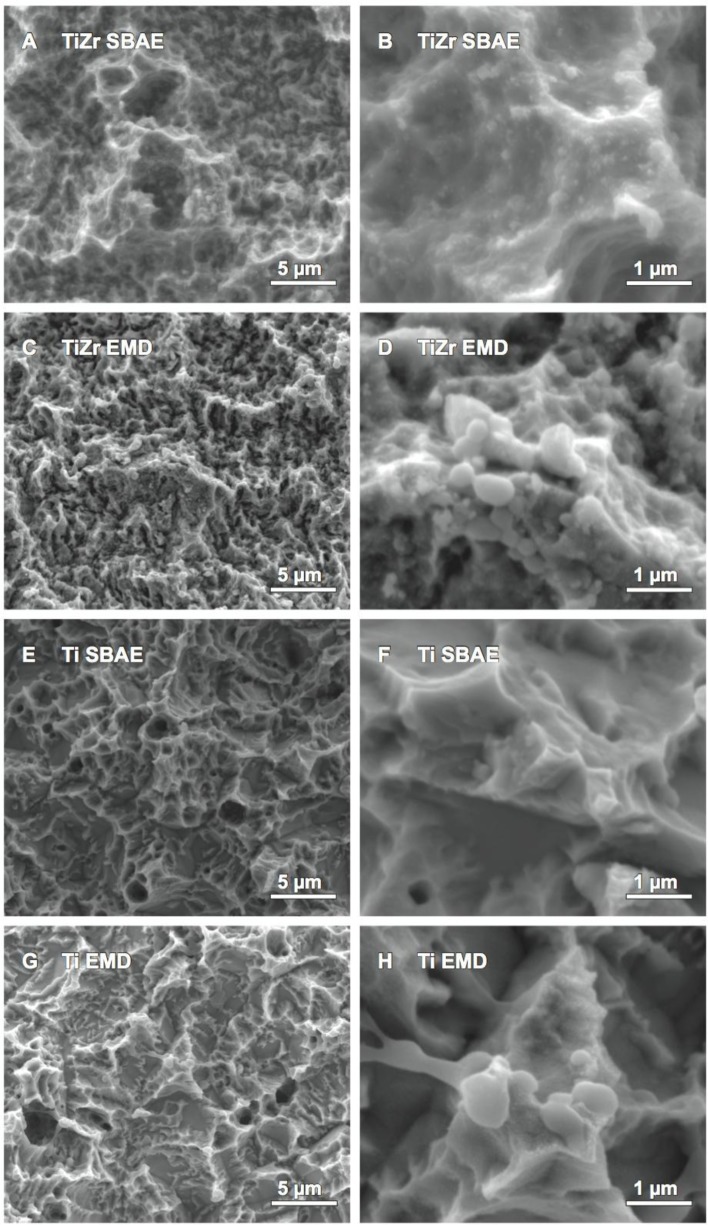
SEM images of (**A**,**B**) TiZr SBAE; (**C**,**D**) TiZr EMD; (**E**,**F**) Ti SBAE and (**G**,**H**) Ti EMD.

Comparison of TiZr EMD against TiZr SBAE by SEM showed changes to the topography for the micro- and nano-topography. Although the micro-topography of SBAE and the coated samples (Figure 2A,C) revealed similar nano-nodules and small spherical structures on the surface, only the EMD coated sample presented additional larger spherical structures that were not visible for the SBAE sample. At larger magnifications (Figure 2B,D) these larger spherical structures, ranging from 70 nm to 650 nm in diameter, appeared to be attached to the surface and showed interconnections to each other. Although, these spherical structures appeared to attach preferably to the peaks of the surface, they were visible on the side faces of the surface peaks as well. The polarization process did not show any other changes to the surface’s topography as the edges and peaks appeared to have the same morphology after polarization as they had prior to polarization. Ti SBAE revealed a similar micro-topography of the surface as it was observed for TiZr SBAE (Figure 2A,E), however the surfaces differed at the nano-level (Figure 2B,F). While TiZr SBAE exhibited nano-nodules and spherical structures, none of the two could be observed for Ti SBAE. By contrast, Ti EMD (Figure 2G) showed spherical microstructures that were of the same size as the spherical structures observed for TiZr EMD at larger magnification (Figure 2H). The structures appeared to preferably cover the peaks and edges of EMD-coated surfaces. Apart from the spherical structures described, there were no other changes of the surface topography observed.

Assessment of the surface micro-topography by optical imaging profilometry only revealed significant changes (*p* = 0.013) against the original SBAE surface for the S_a_ of TiZr EMD (Table 4). The S_a_ was 0.08 μm less rough for TiZr EMD than for TiZr SBAE. There appeared to be a general difference between the two materials, regardless of the surface treatment, as S_a_, S_sk_ and S_ci_ revealed higher values for TiZr, and S_ku_ and S_dr_ revealed higher values for Ti.

### 2.3. Assessment of EMD’s Integrity

**Figure 3.**
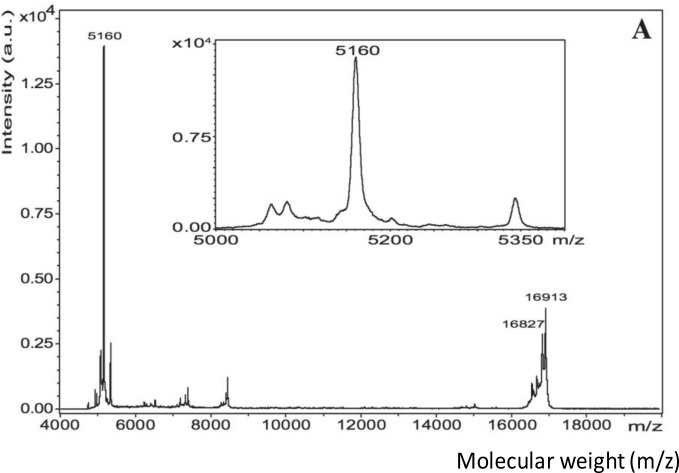

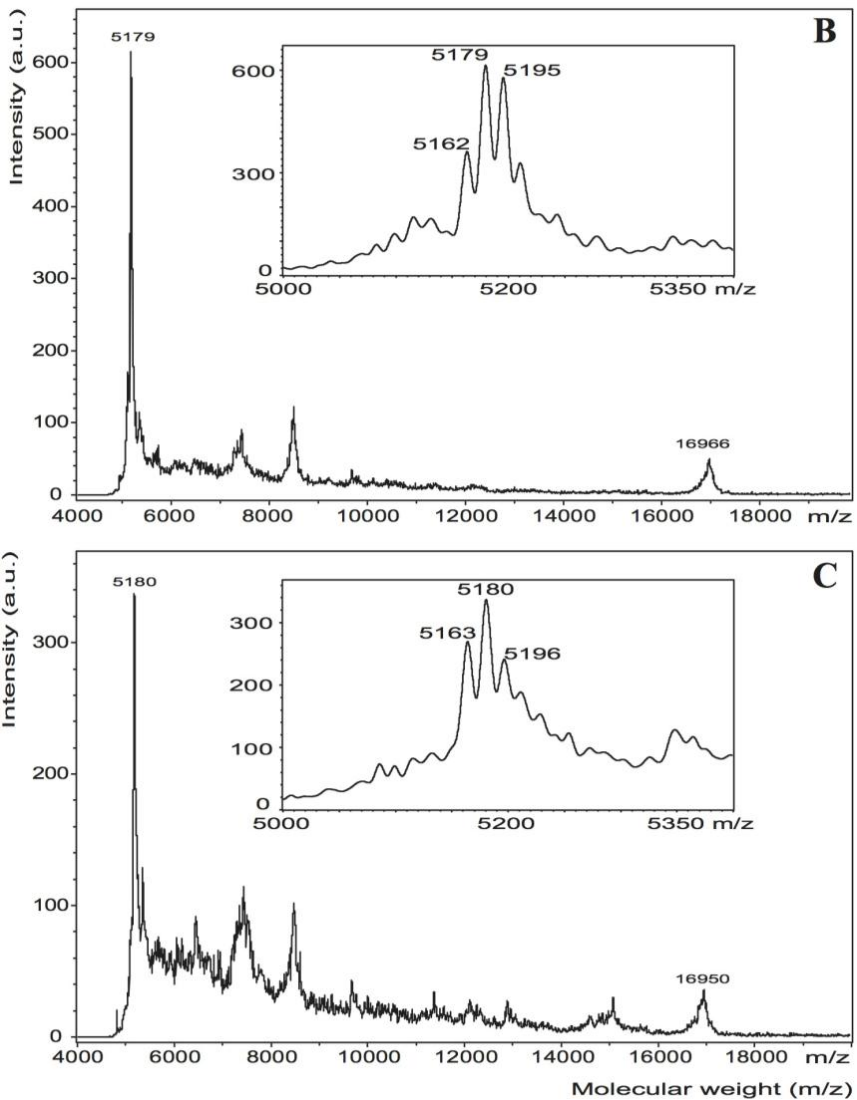
MALDI spectra of (**A**) pure EMD; (**B**) TiZr EMD and (**C**) Ti EMD. All graphs include an enlarged version of the region between 5000 and 5400 m/z.

**Figure 4.**
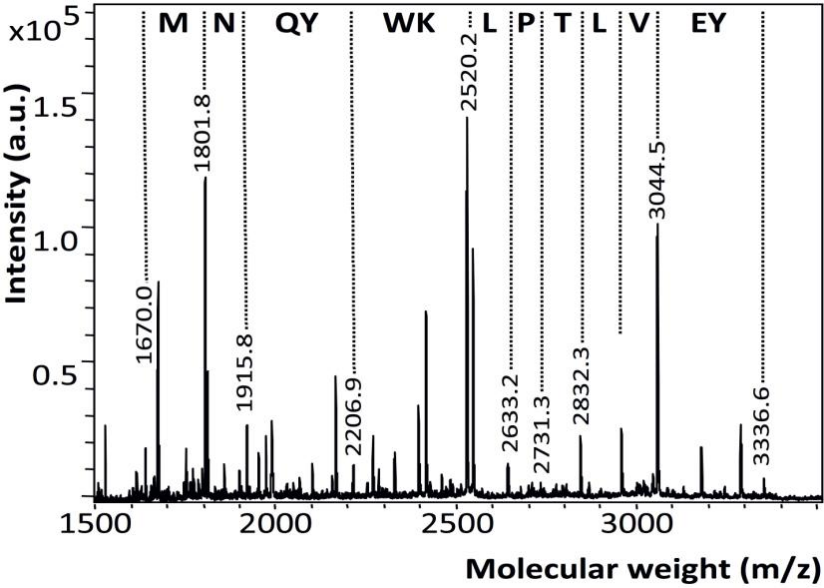
MALDI-ISD of EMD revealed fragments with the sequence YEVLTPLKWYQNM corresponding to the amino acid stretch 33–45.

The matrix-assisted laser desorption/ionization time-of-flight mass spectroscopy (MALDI-TOF MS) spectrum of pure EMD in 0.1% acetic acid revealed a very intense peak in the 5 kDa region and a less intense peak at 16.9 kDa (Figure 3A). Matrix-assisted laser desorption/ionization in source decay (MALDI-ISD) of pure EMD revealed fragments with the sequence YEVLTPLKWYQNM (Figure 4). TiZr EMD showed a major peak at 5179 Da (~620 a.u.) (Figure 3B). This peak was surrounded by peaks with a mass difference of 16 Da. Ti EMD showed its major peak at 5180 Da (~330 a.u.) (Figure 3C). As has been observed for TiZr EMD, this peak was also surrounded by peaks with a mass difference of 16 Da and 17 Da. Moreover, Ti EMD also revealed a peak at 16.9 kDa.

### 2.4. EMD Bioavailability Assessment

**Figure 5.**
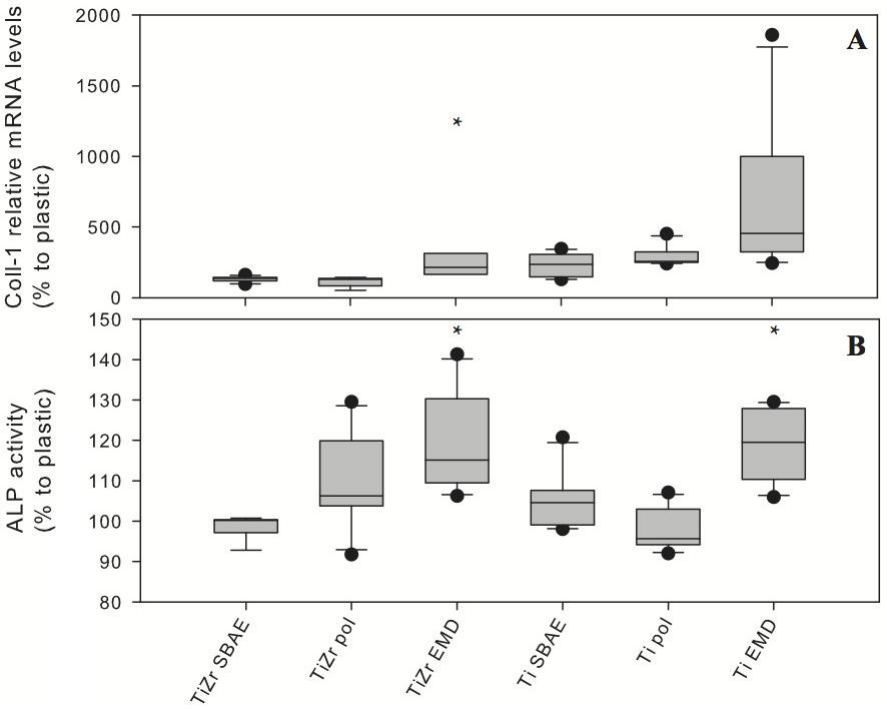
Coll-1 relative mRNA levels (**A**) and ALP activity for polarized only and EMD coated groups were displayed as box plots of the median values (Q2) with 5, 25 (Q1), 75 (Q3) and 95 percentiles (*n* = 6). Student’s t-test revealed significant (* *p* ≤ 0.05) differences in Coll-I relative mRNA levels between TiZr EMD and TiZr SBAE. ALP activity revealed significant differences between TiZr EMD and TiZr SBAE and between Ti EMD and Ti SBAE.

Electro-coated TiZr EMD displayed statistically significant differences (*p* < 0.05) for the expression of Coll-1 mRNA levels (Figure 5A) and alkaline phosphatase (ALP) activity (Figure 5B) compared to TiZr SBAE. Like for TiZr EMD, ALP activity was significantly different for Ti EMD compared to Ti SBAE. By contrast, the expression of Coll-1 mRNA levels of Ti EMD was not significantly higher compared to Ti SBAE, although higher gene expression was observed. The pol groups did not expose any significant differences in gene expression compared to TiZr SBAE.

## 3. Discussion

### 3.1. EMD Bioavailability Assessment of the Coated Surfaces

TiZr EMD exhibited earlier proliferation than TiZr SBAE, concluding from the significantly increased expression of Coll-1 and ALP activity based on the temporal expression presented by Quarles *et al.* [48] and Monjo *et al.* [49]. Likewise, Ti EMD tended towards earlier cell proliferation than Ti SBAE, based on significantly higher ALP activity and a trend towards increased expression of Coll-1, although this difference was not statistically significant. As TiZr pol and Ti pol did not show any significant differences to the performance of the respective SBAE group, the enhanced cell proliferation was linked to the EMD-coating of the surface and confirmed that EMD was not only bio-available but also maintained its function. The findings presented for EMD-coated surfaces agreed with the results reported by Reseland *et al.* [50], who also showed increased Coll-1 levels and ALP activity for primary human osteoblasts incubated with EMD. A different study by Rubert *et al.* [51] has also shown increased Coll-1 mRNA levels after 14 days for MC3T3-E1 cells incubated with EMD. A recent *in vitro* study using rat calvarial osteoblasts by Miron *et al.* [43] assessed a titanium SLA^®^ surface (Straumann, Basel, Switzerland), similar to the Ti SBAE surface used in this study, that was coated with EMD by a dipping procedure. Their study showed enhanced cell proliferation and cell differentiation for EMD-modified Ti SLA^®^. Miron *et al.* [43] concluded that EMD accelerated differentiation by promoting mature phenotypes earlier than Ti alone.

### 3.2. Confirmation of EMD’s Integrity after Coating

As EMD consists of a multitude of components of different size and mass, MALDI was used to analyze the individual fractions of an EMD-coated surface in order to confirm the integrity of the coating. The MALDI spectra of TiZr EMD and Ti EMD showed peaks in the region of 5 kDa and 16 kDa comparable to the peaks found for pure EMD. The minor differences in peak mass observed were not unusual when comparing it to the results of other authors [52,53]. Mumulidu *et al.* [52] reported a similar range for the 5 kDa peak of EMD when analyzing this peak specifically. They concluded that the mass variation observed for this peak might be a result of the small amount of the sample itself [52]. Moreover, this study showed the major peak in the 5 kDa region to be surrounded by peaks with a mass difference of +16 Da that were corresponding to oxidation. The peak found at 16.9 kDa for pure EMD was also observed for TiZr EMD and Ti EMD. Riksen *et al.* [54] showed a peak at 16.6 kDa for two different amelogenin fractions separated from EMD by size-exclusion high-performance liquid chromatography. As Riksen *et al.* [54] used 0.05% acetic acid for dissolving EMD, the pH should have been slightly higher than for the 0.1% acetic acid solution used in this study. Cohen *et al.* [55] described a pH dependency of the mass peak of a MALDI measurement when using a HCCA matrix like it was used in this study. While pure EMD was dissolved in 0.1% acetic acid at pH 3.1, the ACN+TFA solution used for detaching EMD from the coated coins had a pH of 1.8. It was believed that the results obtained by Riksen *et al.* [54] were measured at a pH that was different to the pH used for the samples of this study. Hence, an influence of the different pH values of the single samples could not be excluded. Even though EMD consists of a multitude of components of different size and weight, a successful coating with EMD was supported by MALDI as the fractions found on EMD coated surfaces largely corresponded with the fractions observed for pure EMD.

The peaks observed in the MALDI spectrum of pure EMD in the 5 kDa and 16 kDa region concurred with the peaks that have previously been reported in the literature [40,52–54]. Moreover, sequencing of pure EMD MALDI-ISD revealed fragments with the sequence YEVLTPLKWYQNM that were corresponding to the amino acid stretch 33–45. Considering the detected molecular mass at 5158 Da and the determined sequence, this molecule corresponded to amelogenin, which is the major component of EMD, sequence stretch 17–59 without the signal peptide and with an additional mass of 80 Da within sequence stretch 17–32 [40,53]. This mass difference indicated towards a phosphorylation. Notably, serine-32 has been identified to be phosphorylated in bovine amelogenin [53].

### 3.3. Chemical Confirmation of an Effective Surface Coating with EMD

The increased carbon layer thickness and total carbon content for TiZr EMD and Ti EMD observed by SIMS suggested an incorporation of EMD into the surface. Detailed analysis of the coated surfaces by XPS showed a shift in the carbon binding state on the outer surface towards a binding state distribution of C–C, C=C, and C–O/C–N bonds that was comparable to the distribution observed for pure EMD. Moreover, surface oxygen was significantly decreased for EMD-coated samples while the oxygen binding state was shifted from Ti-bond oxygen towards organically bound oxygen, which correlated with the binding state of pure EMD, which has about 80% organically bound oxygen. In addition, EMD related nitrogen, silicon, and sulfur that have not been detected on the initial SBAE surfaces could be observed for EMD-coated surfaces. The decrease in Ti and Zr levels on the surface for EMD-coated samples indicated that the surface was masked by the EMD-coating like it has been described by Morra *et al.* [56] for a different surface coating.

The presence of fluoride on SBAE and EMD surfaces was not intended and was believed to have derived from handling of the samples with PTFE covers during blasting, etching and polarization. The presence of Na and Cl was believed to be a result of the storage of the SBAE samples in saline solution. Even though the SBAE samples were washed in reverse osmosis deionized water in an ultrasonic bath, for 5 min, prior to examination or polarization, an adhesion of NaCl to the respective surface remained possible and was most likely the source of these trace elements. The results observed for the SBAE surfaces were in accordance with previous studies on similar surfaces [56,57].

The hydride layer created by this process was proposed to be the linking element between the metal surface and the biomolecule. Therefore, the hydrogen depth profile was of particular interest. The results of this study showed increased hydrogen maximum hydrogen intensity, total hydrogen content, and layer thickness of the EMD-coated samples when compared to the corresponding SBAE samples. This was in agreement with the coating mechanism that has been suggested by Lyngstadaas and Ellingsen [35].

### 3.4. Visual Confirmation of Effective Surface Coating with EMD

The results of the SEM analysis showed large interconnected spherical structures for TiZr EMD and Ti EMD that were not present on the respective SBAE surface. Interestingly, the structures observed for EMD-coated samples were different to the structures observed in our previous study that used the same polarization process without any biomolecules on the same SBAE materials [37]. While the structures observed for polarized SBAE surfaces in our previous study were shaped differently, they also seemed to have grown from inside the material towards the outer surface. By contrast, the spheres observed for EMD-coated samples in this study appeared to have been attached on top of the material from the surroundings. The masking effect of titanium and zirconium respectively previously described supported this observation. Such an attachment would be in concordance with the attachment of EMD to the surface as it was suggested. Moreover, Gestrelius *et al.* [58,59] showed EMD precipitated from aqueous solution to form spheres or short rods that were comparable to the structures observed for EMD-coated samples in this study. Thus, the spheres and short rods seen on the surface of EMD coated samples in the SEM images of this study were interpreted to be EMD.

Analysis of the surface roughness parameters by optical imaging profilometry did not reveal significant changes after EMD-coating except for the S_a_ of TiZr EMD. Even though the change was statistically significant, the actual S_a_ of TiZr EMD was midway between the S_a_ of TiZr SBAE and Ti SBAE. The observed trend towards generally different surfaces for TiZr and Ti has been reported in previous studies [10,57]. It has been shown in *in vivo* studies that TiZr SBAE performed equally well if not better than Ti SBAE [60–62]. Moreover, S_a_ values between 1.16 and 3 μm have been shown to optimal for titanium endosseous dental implants with a surface comparable to the SBAE surface [63–65]. Hence, the surface topography created during EMD-coating should not have a negative effect on the performance of the surface but an effect may only be expected from the coating itself.

## 4. Experimental Section

### 4.1. Samples

This study used coin-shaped samples made of grade IV commercially pure titanium (Ti) and a titanium-zirconium alloy (TiZr) containing 13% to 17% zirconium with a grit-blasted and acid-etched (SBAE) surface with a diameter of 4.39 mm and a height of 2 mm. Detailed information about the samples have been described in our previous study [10] and in other studies [13,20]. The setup used for cathodic polarization was the same as it has been described in our previous study [37]. Polarization was performed in 200 mL of a 2 M buffer solution mixed of acetic acid and sodium acetate at pH 5. 20 mg of dry stored EMD (Institut Straumann AG, Basel, Switzerland) were dissolved in 2 mL of 0.1% acetic acid at 4 °C and added to the buffer for final EMD-concentration of 0.01 mg/mL. The buffer was kept at 21 °C over the course of the whole process. After processing, all samples were air-dried in a laminar flow cabin for 30 min and packed in Eppendorf tubes. The coating of TiZr SBAE with EMD (TiZr EMD) was done for 60 min while the output current was set to 0.49 mA/cm^2^. Ti SBAE was coated with EMD (Ti EMD) for 60 min at a current density of 1.65 mA/cm^2^. A control group that was cathodically polarized without addition of EMD to the buffer (TiZr pol; Ti pol) was added to the cell study to assess the effect of the polarization independently of EMD coating. The parameters were chosen for the particular materials based on the results of our previous study that showed a promising development of the hydride levels for attaching charged biomolecules for those settings [37].

## 4.2. Chemical Characterization

TiZr SBAE samples and EMD-coated samples were analyzed by SIMS in order to demonstrate EMD incorporation into the surface by assessment of the ^12^C isotope’s depth profile, as EMD consists to a large part of carbon. Lamolle *et al.* [9] used this method to show the incorporation of fluorine into the surface of titanium after etching in hydrofluoric acid. Furthermore, depth profiles of the ^1^H and ^18^O isotopes were obtained. Analysis was performed on an IMS 7f (Cameca, Paris, France) magnetic sector SIMS using the same parameters that have been described in our previous study [10].

Analysis of the surface by XPS was used to detect changes in the surface chemistry and binding states after polarization with EMD. The XPS analysis was carried out on an Axis Ultra^DLD^ XP spectrometer (Kratos Analytical Limited, Manchester, UK). Detail spectra were recorded for O 1s and C 1s. The energy shift due to surface charging was below 1 eV based on the C 1s peak position relative to the established BEs, therefore the experiment was performed without charge compensation. All other settings of the instrument were the same that have been described in our previous study [10]. Peaks were interpreted according to Moulder and Chastain [66], and Cai *et al.* [67].

### 4.3. Surface Morphology

All SEM images in this study were taken on a Quanta 200 FEG (FEI Hillsboro, OR, USA) field-emission SEM. Its Schottky field emission gun (FEG) allowed high spatial resolution. All samples were sputtered with platinum for one minute prior to imaging and mounted on the sample holder with conductive carbon tape.

A PLμ 2300 (Sensofar-Tech S.L., Terrassa, Spain) blue light laser profilometer and interferometer using a 50× EPI (Nikon, Tokyo, Japan) confocal objective was used for assessing the surface topography. The following surface parameters were analyzed: average roughness (S_a_), skewness of the height distribution (S_sk_), kurtosis of the height distribution (S_ku_), core fluid retention index (S_ci_), and developed interfacial area ratio (S_dr_).

### 4.4. Analysis of the Biomolecules

As EMD is almost insoluble at physiological pH and temperature, it was detached from the surface in a solution containing 40% acetonitrile (ACN) and 0.3% trifluoro acetic acid (TFA) at pH 2 and at 8 °C [40]. Samples were submerged in 1 mL of ACN+TFA and placed on a shaker for 24 h. Pure EMD was dissolved in acetic acid as described earlier. All samples were cleaned using a C4-ZipTip to remove impurities that may have created background noise during MALDI-TOF MS. 0.5 mL of 20 mg/mL α-Cyano-4-hydroxycinnamic acid (HCCA) matrix in 0.3% TFA/acetonitrile (2:1) was added to each sample before spotting it onto a stainless steel MALDI plate. MALDI-TOF MS was performed on an ULTRAFLEX II MALDI-TOF/TOF (Bruker Daltonics, Bremen, Germany). Basic settings of the instrument for the spectra were as follows: ion source 1: 25 kV; ion source 2: 23.5 kV; lens: 6.5 kV; deflection mass 4000 Da, polarity positive. Basic setting for MALDI-in source decay (MALDI-ISD) were as follows: ion source 1: 25 kV; ion source 2: 21.85 kV; lens: 9.7 kV; reflector: 26.3 kV; reflector 2: 13.85 kV; deflector mode, polarity positive. FlexControl 3.0 (Bruker Daltonics, Bremen, Germany) was utilized for data acquisition and FlexAnalysis 2.4 (Bruker Daltonics, Bremen, Germany) for further analysis.

### 4.5. Bio-Availability Study

An *in vitro* cell study was performed to evaluate the bioavailability of the biomolecule on the coated surfaces by comparing the EMD-polarized samples against the respective SBAE surface. All groups had a group size of six samples per group. A group that was only polarized without any EMD in the buffer was used as an additional control group. The murine osteoblastic cell line MC3T3-E1 was obtained from the German Collection of Microorganisms and Cell Cultures (DSMZ, Braunschweig, Germany). All experiments were performed in the same passage of the MC3T3-E1 cells (passage 16). The same number of cells was cultured in parallel on plastic for all experiments and the results were presented as relative to the expression of the plastic control group in percent. The detailed steps for cell culturing, used housekeeping genes and analytical methods for RNA isolation, real time polymerase chain reaction analysis and determination of ALP activity were performed as it has been described by Monjo *et al.* [49]. ALP activity and Coll-1 mRNA levels were assessed after 14 days.

### 4.6. Statistical Analysis

Data were compared by a two way ANOVA in SigmaPlot 11 (Systat Software, San José, CA, USA). A normality test was performed; once this was passed, all samples were compared in pairs using the Holm-Sidak method. ANOVA was performed on ranks when the normality test failed, using the Student-Newman-Keuls test or Dunn’s test for pairwise comparison. Significance levels were set to significant * *p* ≤ 0.05 and highly significant ** *p* ≤ 0.01. All data were displayed as arithmetic mean values with standard deviation when the data were distributed normally and as median values with interquartile range when the data were not distributed normally. The results of the cell study were compared by a paired student’s t-test and displayed as box plots of the median values (Q2) with the 5, 25 (Q1), 75 (Q3), and 95 percentiles.

## 5. Conclusions

Cathodic polarization under acidic conditions can be used to coat commercial implant surfaces with the growth-promoting agent EMD. Moreover, the coated surfaces revealed intact EMD that was bio-available and maintained its function. The hydride layer of the original SBAE surfaces could be activated by the process as an intermediate stage of charged hydride that acted as a coupling layer for the biomolecule. The coating presented in this study can be applied in an integrated manner of a one-step process that does not require modifications or pre-treatments of the commercially available base material prior to coating and is gentle enough to not inactivate or degrade protein amino acid-based biomolecules. Although the current study only assessed Ti and TiZr with SBAE surfaces, the process should be transferable to other metallic materials that develop a hydride layer during cathodic polarization and may potentially be used for other biomolecules that ionize under acidic conditions as well.

## Figures and Tables

**Table 1.** SIMS depth profile analysis parameters for the ^1^H and ^12^C isotope.

Isotope	Sample	Total amount (c)	Maximum intensity (c/s)	Layer thickness (μm)
^1^H	TiZr SBAE	2.65 × 10^7^	6.05 × 10^7^	2.26
^1^H	TiZr EMD	5.67 × 10^7^	1.28 × 10^8^	2.92
^1^H	Ti SBAE	5.44 × 10^6^	1.47 × 10^8^	1.1
^1^H	Ti EMD	2.10 × 10^7^	1.07 × 10^8^	1.3
^12^C	TiZr SBAE	3.61× 10^4^	2.42 × 10^5^	0.06
^12^C	TiZr EMD	2.76 × 10^5^	5.94 × 10^6^	0.75
^12^C	Ti SBAE	1.53 × 10^5^	1.29 × 10^6^	0.03
^12^C	Ti EMD	3.37 × 10^6^	1.02 × 10^7^	1.16

**Table 2.** XPS surface elements distribution.

Element	Pure EMD (%)	TiZr SBAE (%)	TiZr EMD (%)	Ti SBAE (%)	Ti EMD (%)
O 1s	24.69	53.79	19.58	54.24	23.64
C 1s	60.91	20.74	62.55	22.74	56.66
F 1s	–	0.78	–	1.29	0.87
Ti 2p	–	19.45	1.91	21.73	2.91
N 1s	13.90	–	12.63	–	8.64
Cl 2p	–	1.55	0.59	–	0.69
Na KLL	–	0.78	1.27	–	5.76
Si 2p	0.15	–	0.92	–	0.83
S 2p	0.36	–	0.17	–	–
Zr 3d	–	2.90	0.37	–	–

**Table 3.** XPS specific bindings of the surface oxygen and carbon.

Element	Assignment	Pure EMD		TiZr SBAE		TiZr EMD		Ti SBAE		Ti EMD	

		Position (eV)	Conc. (at%)	Position (eV)	Conc. (at%)	Position (eV)	Conc. (at%)	Position (eV)	Conc. (at%)	Position (eV)	Conc. (at%)
O 1s	Organic O	531.16	80.62	531.11	35.35	531.44	85.00	531.19	35.71	531.43	89.04
O 1s	TiO_2_	–	–	529.94	64.65	529.24	15.00	529.97	64.30	529.09	10.96
C 1s	C–C, CH*_x_*	284.75	52.72	284.71	56.95	284.69	47.94	284.69	59.17	284.73	51.31
C 1s	C=C	287.92	20.81	288.63	10.35	287.83	20.94	288.81	14.38	288.05	25.65
C 1s	C–O, C–N	286.05	26.47	286.10	32.70	285.95	31.12	286.10	26.44	285.93	23.04

**Table 4.** Mean surface topography parameters with standard deviation assessed by optical imaging profilometry. Only TiZr EMD showed a significant difference (* *p* < 0.05) for S_a_ compared to TiZr SBAE.

Sample	S_a_ (μm)	S_sk_	S_ku_	S_ci_	S_dr_ (%)
TiZr SBAE	2.074 ± 0.08	−0.142 ± 0.07	2.920 ± 0.14	1.530 ± 0.03	58.19 ± 2.14
TiZr EMD	1.904 ± 0.06 *	−0.146 ± 0.09	2.939 ± 0.05	1.528 ± 0.03	58.78 ± 5.06
Ti SBAE	1.861 ± 0.12	−0.195 ± 0.35	3.800 ± 1.07	1.433 ± 0.09	70.51 ± 4.94
Ti EMD	1.816 ± 0.04	−0.359 ± 0.13	3.641 ± 0.81	1.448 ± 0.05	72.51 ± 3.21
